# Structural Basis of Lipopolysaccharide O-Antigen Chain Length Modality

**DOI:** 10.34133/research.1276

**Published:** 2026-05-12

**Authors:** Benjamin Wiseman, Göran Widmalm, Martin Högbom

**Affiliations:** ^1^ Department of Biochemistry and Biophysics and Science for Life Laboratory, Stockholm University, Stockholm, Sweden.; ^2^ Department of Chemistry, Stockholm University, Stockholm, Sweden.

## Abstract

Lipopolysaccharides are important components of the gram-negative bacterial cell envelope that are involved in immune evasion and act as a protective barrier. Employing cryo-electron microscopy, we resolved the structure and dynamics of FepE, the copolymerase component of the Wzy-dependent pathway, responsible for the length modulation of very long O-antigen molecules. Comparison of the interior volumes of related copolymerases’ periplasmic domains with the volume of hydrated sugars suggests that the size of the periplasmic domain controls the length of the O-antigen, implying that polysaccharide chain polymerization occurs inside the copolymerase periplasmic domain. Moreover, we show the opening of the FepE complex as well as other large mechanistically relevant movements. The opening of the complex presents an attractive corridor for the release of completed polysaccharide chains.

## Introduction

Lipopolysaccharides (LPSs) are important components of the outer membrane of gram-negative bacteria that play essential roles in many biological processes such as biofilm formation, resistance to bile salts, and immune evasion and act as a protective barrier against the environment and toxic molecules such as antibiotics [1,2]. LPS molecules are made of a lipid A anchor, a core oligosaccharide (OS), and a polymer of repeating units (RUs) of sugars that defines the O-antigen (Oag) serogroup [1,3]. Most Oags are made via the Wzy-dependent biosynthetic pathway where they are polymerized directly at the inner membrane [4]. In this pathway, Oag synthesis is started in the cytoplasm with the sequential glycosyltransferase-mediated addition of monosaccharides to a monosaccharide-PP-undecaprenyl acceptor, resulting in an OS linked to the lipid anchor. This OS-PP-Und entity is then flipped across the inner membrane by the Wzx flippase. Once flipped, the OSs are polymerized by the Wzy polymerase and the Oag chain length is modulated by the Wzz copolymerase [2,4,5]. The Oag is subsequently ligated to a lipid A-core molecule by the WaaL ligase [6] to form the LPS molecule that is shuttled to the outer membrane via the Lpt system [7,8].

It has been suggested that the LPS chain length has been optimized for, among other things, survival during host infection [9,10]. The lengths of the Oag chains that are modulated at the bacterial inner membrane typically fall into distinct modal distributions, classically categorized as “long” and “very long”. Depending on the bacterial species, strain, and environmental conditions, “long” Oags contain 16 to 35 RUs [5,11] and in *Escherichia coli* can range from 16 to 25 RUs [12]. Very long Oags, on the other hand, can contain >80 to >100 RUs [5,13], with those in *E. coli* consisting of more than 80 RUs [14,15]. Polysaccharide copolymerases modulate the length distribution of the synthesized polysaccharide chain through likely interacting with the membrane-bound polymerase Wzy as it polymerizes the growing Oag polysaccharide. These integral membrane proteins are known to homo-oligomerize into distinctive structures containing a large conical frustum-like periplasmic domain with N- and C-terminal transmembrane domains encircling a large chamber [16–18]. In fact, many bacteria contain 2 copolymerases that can modulate long and very long Oags: WzzB and FepE, respectively. However, despite several decades of intense structural and biochemical analysis and several potential mechanisms suggested [4,5], a molecular mechanism of LPS polymerization and chain length modulation has so far remained elusive. Complicating the issue, seemingly contradictory studies [14,15,19–22] have suggested that residues on both the interior and exterior of the periplasmic domain are responsible for modulating the chain length of the growing polysaccharide. However, recent successes in structure determination of near-full-length polysaccharide copolymerase complexes [16,17,23–25] using single-particle cryo-electron microscopy (cryo-EM) are beginning to shed new light on this essential biological process.

**Fig. 1.**
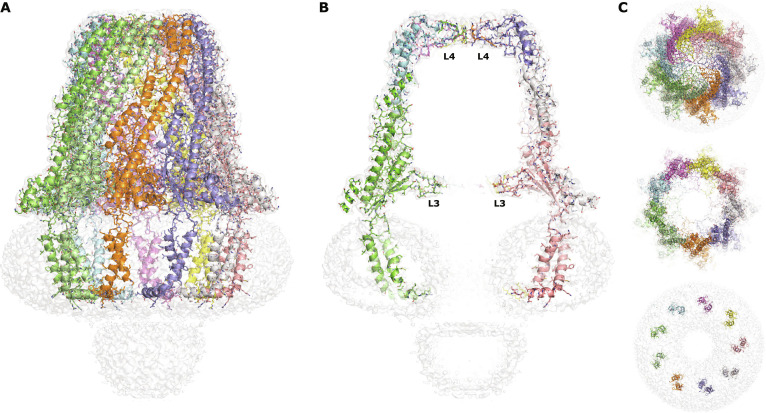
Overall structure of a closed nonameric *Escherichia coli* FepE complex. (A) Cartoon representation of the closed nonameric FepE complex overlaid with the C9-symmetrized FepE cryo-electron microscopy (cryo-EM) density map (white). (B) Sliced to display the interior of the complex. (C) Top view looking down from the periplasmic side. Top: view looking down from the top of the periplasmic domain. Middle: sliced to the level of the interior periplasmic L3 loop. Bottom: sliced to the level of the membrane to display the doughnut-shaped detergent micelle (white) and 9 transmembrane domains.

With that in mind, using single-particle cryo-EM (Figs. S1 and S2), we determined the structure of FepE, a polysaccharide copolymerase responsible for the modulation of very long LPS molecules. We show that FepE forms a closed nonameric complex with a large barrel-like periplasmic domain (Fig. 1), in contrast to octameric complexes as seen with other members of this family. Our analysis reveals that adjustments to loops and α-helices located along the interior wall contribute to a barrel volume roughly 3.6 times larger than that of WzzB, despite similar overall dimensions. By comparing the periplasmic volumes with that of the volume of hydrated sugars, we demonstrate that polysaccharide chain length modulation is likely controlled by the internal volume size of the polysaccharide copolymerase’s periplasmic domain, and therefore, polymerizing Oag molecules must grow along the interior of the periplasmic domain. Additionally, we found multiple oligomeric states of FepE in open conformations (Fig. S3), and utilizing principal component analysis, we are able to directly visualize from the cryo-EM data, as well as other movements, the opening of the nonameric complex presenting an attractive avenue for full-length Oag release.

## Results

### FepE contains a large barrel-shaped periplasmic domain interior

**Fig. 2.**
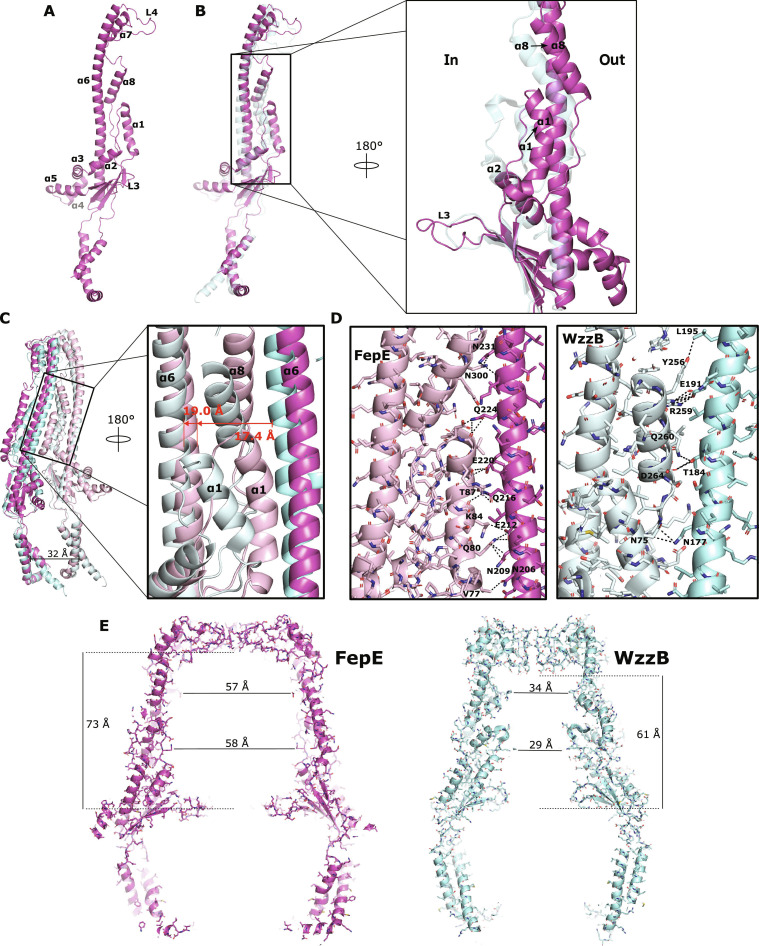
Structural comparison of the O-antigen *Escherichia coli* polysaccharide copolymerases. (A) Cartoon representation of a single FepE protomer with labels of the α-helices. (B) Structural alignment of the single protomers of WzzB (Protein Data Bank [PDB] ID 6RBG) (cyan) to those of FepE (magenta). Boxed: zoom of the region around α-helices 1 and 8 showing their repositioning compared to that in WzzB. In and Out refers to the interior and exterior of the periplasmic domain, respectively. (C) Protomer packing comparison between FepE (magenta) and WzzB (cyan). The repositioning of α1 into a more vertical position in FepE results in an increase in the distance of α6 between protomers. (D) Interacting residues of α1 involved in protomer packing. Due to the repositioning of the α1 of FepE, 6 residues of α1 interact with the adjacent α6 compared to only 1 residue in WzzB. (E) Sliced to display the interior of the periplasmic domains. The repositioning of the α-helices results in a near doubling of the interior dimensions of FepE.

Overall, the FepE periplasmic domain is similar to the previously described structures: a large domain ~100 Å in height above the membrane interface that contains a hollow interior (Fig. 1). The characteristic frustum-shaped complexes of this family arise from a side-by-side packing of protomers, and the interactions observed here are similar to what have been described elsewhere: α1, α7, and α8 of one protomer interacting with the long α6 of an adjacent protomer between protomers of FepE and WzzB. However, at the protomer level, there are important differences (Fig. 2 and Fig. S4). Located along the interior wall of the periplasmic domain, FepE’s α1 and α8 are shifted backward toward the exterior when compared to those of WzzB (Fig. 2B). While α8 simply translates backward by roughly 8.5 Å, α1 translates backward by 7.0 Å and tilts by 35° to be nearly parallel to the long α6 helix. This shifting and tilting to a nearly parallel position also results in the small loop connecting α1 and α2 to be pulled backward by 9.3 Å (Fig. 2B), which in turn pulls α2 slightly backward by roughly 4.0 Å when compared to that of WzzB. This can further be visualized by the comparison of the side-by-side packing of protomers between FepE and WzzB (Fig. 2C) with the long α6 between protomers separated by 19.0 and 17.4 Å, respectively. The slightly longer separation distance of 19.0 Å between α6 helices seen in FepE can be explained by both α1 and α8 that are pushed back in between the α6 helices of adjacent protomers. In this position, virtually the whole length of the α1 of one protomer is now interacting with the α6 of the adjacent protomer (Fig. 2D). The slightly shorter separation distance of 17.6 Å between α6 helices in the protomers of WzzB likely squeezes the α1 helices into tilting toward the interior of the WzzB bell, resulting in interactions with only its N-terminus with the α6 of an adjacent protomer (Fig. 2D). Similarly, the pushing back of α8 seen in FepE results in the interaction of its N-terminus with the neighboring α6 compared to interactions across the entire helix as seen in WzzB. The N-terminal region of α8 has been shown to be important in protein stability and oligomerization [14,21,26].

Although these differences seem minor at the protomer level, they have a dramatic effect on the interior dimensions of the periplasmic domain of the full nonameric FepE complex (Fig. 2E). The pushing back and shifting vertical of these loops and helices against the main α6 of each protomer around the periplasmic domain creates a smooth interior wall of the FepE complex. The homologous loops and helices of WzzB, on the other hand, are pointed inward toward the center of the octameric complex, substantially constricting the internal space of the periplasmic domain. This results in a dramatic near doubling of the internal diameter of the FepE periplasmic domain, converting the periplasmic bell seen in WzzB into a large barrel. Additionally, in order to maintain a closed barrel due to the much larger internal diameter of FepE, the L4 loops, located at the top of the periplasmic domain and known to be essential for long LPS modulation [14,19,21], are significantly extended compared to those of WzzB. This results in an increased height of the internal cavity by raising the ceiling of the periplasmic domain by more than 10 Å (Fig. 2E). Thus, when added together into the full nonameric complex, these slight modifications to the structure of FepE (the extended L4 loops and the pulling and tilting backward of internal loops and helices) create a massive, barrel-shaped periplasmic domain, with interior dimensions 1.2 times taller and ~2 times wider compared to those of WzzB.

### Open conformations of the FepE complex adopt a split-ring washer conformation

**Fig. 3.**
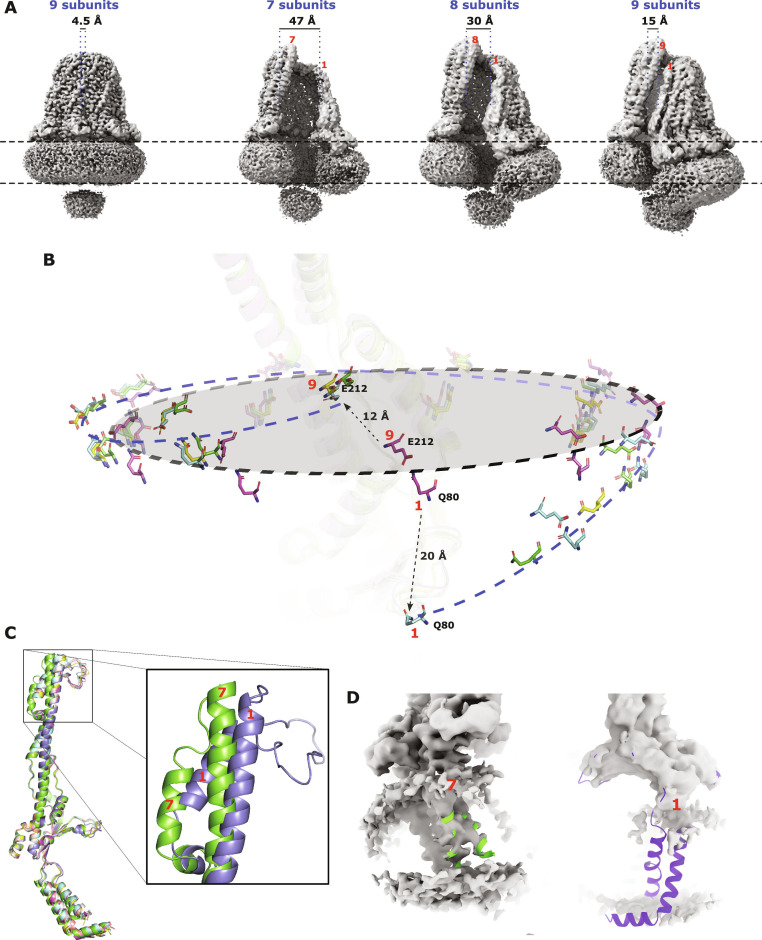
The open states of FepE. (A) Cryo-electron microscopy (Cryo-EM) density maps of the FepE open states compared to the closed nonameric state. Three open states containing 7, 8, and 9 subunits were observed. Compared to the closed state, the open states adapt a split-ring washer conformation. Distances are from the first to the last protomer. The dashed line represents the predicted level plane of the membrane of the closed state. (B) Plot showing the location of the Q80–E212 interaction between the subunits of the 7-, 8-, and 9-protomer open complexes (yellow, green, and cyan, respectively) and the closed (magenta) nonameric FepE. The gray plane and black dashed line represent the level plane of the closed nonameric state. The dashed blue line represents the splitting of the plane as seen in the open states. The states are aligned using stable subunit 5 at the back, opposite to the splitting (transparent cartoon). (C) Representative structural alignment of the individual protomers of the 7-subunit state of FepE. Regardless of the oligomeric state, the open conformation subunits are highly similar with a root mean square deviation (RMSD) between 0.6 and 1.5 Å (Table S2). The main difference across all open states is the slight bending outward of the top of α6 and the repositioning of α7 (zoomed box) between the first and the last protomer. (D) Transmembrane region of the 7-subunit state. The cryo-EM density for the transmembrane domain of the first subunit is missing. The purple cartoon represents its expected location. This trend is seen across all open states. Throughout the figure, red numbers represent subunit number.

In addition to the closed nonameric complex, open complexes containing 7, 8, and 9 FepE protomers were also detected (Fig. 3 and Figs. S1 to S3). This is consistent with the previously reported complex of WzzB [16] determined by the single-particle technique that detected small particles of purified WzzB that could not be accurately measured in addition to the reported octameric complex. Here, by contrast, we were able to isolate and determine the structures of multiple oligomeric states to a relatively high resolution (Fig. S3). It is likely that the additional protomer–protomer interactions described above are providing increased stability to the FepE complex, allowing these open complexes to be isolated. Although the effect of detergent solubilization and overexpression cannot be ruled out, this is consistent with previous cross-linking experiments [21,22,27,28] and structural studies [19,20,27] that suggested that multiple oligomeric states could exist. Consistent with 1 and 2 missing protomers, respectively, the 7- and 8-subunit complexes contain a considerable opening into the periplasmic domain of 47 and 30 Å, respectively, that the missing subunits could easily slot into (Fig. 3A). However, the open 9-subunit complex contains an opening of 15 Å, too small to accommodate an additional subunit, confirming the nonameric nature of the complex. No closed complexes containing 7 or 8 protomers were detected, further supporting a nonameric closed FepE complex. Additionally, no complexes smaller than 7 protomers were possible to isolate.

The open FepE complexes adapt a conformation reminiscent of a split-ring washer (Fig. 3A and B) with the near-rigid-body displacement of entire individual subunits around the complex. The open complexes described here follow the same trend: their tilt increases as they turn away from the reference protomer (subunit 5, transparent cartoon in Fig. 3B). The open 9-subunit complex has the most extreme tilt, followed by the 8- and 7-subunit complexes. Additionally, when compared to the closed complex, turning clockwise from protomer 5, the complex tilts below the plane, and while turning counterclockwise from protomer 5, the complex tilts above the plane (Fig. 3B), creating the split-ring washer effect. Regardless of the oligomeric state, the main difference between protomers within an open complex is the slight bending backward of the main α6 and the repositioning of α7 at the top of the periplasmic barrel (Fig. 3C, Fig. S3, and Table S2) and the lack of cryo-EM density of the L4 loop of this protomer. This distortion is remarkably similar to the alternating subunits seen in WzzE [17], suggesting that this bending could be a common theme across this family of proteins. Similarly, the lack of density for this protomer’s L4 loop is consistent with the presumed flexibility that, despite being located far from the membrane interface, is vitally important in the production of long polysaccharide chains [14,18,19,21]. Estimations of local resolution (Fig. S2) show significantly lower resolutions of the end protomers of the open states (for example, protomers 1 and 9 of the nonameric complex), consistent with them lacking the stabilization of a second adjacent protomer. Additionally, in all 3 open complexes, cryo-EM density for the transmembrane domain of the first protomer is completely missing, suggesting that this region is extremely dynamic in nature (Fig. 3D).

The split-ring conformation seen in the open FepE complexes is striking and could suggest that the FepE complex could contribute to the deformation of the *E. coli* inner membrane. Although the effects of detergent solubilization cannot be ruled out, there are multiple examples of large membrane protein complexes that can cause membrane deformation [29,30]. For example, the split-ring open FepE conformations look remarkably similar to recent structures of the membrane attack complex [31,32] of the innate immune system. Many factors are known to work alone or in tandem to create membrane deformation in biological systems [33]. These include the asymmetry of lipid organization and composition created by flippases, which unidirectionally flip lipids across a bilayer, as well as lipid modification and clustering. Additionally, high local concentrations of proteins on a membrane surface can also cause deformation [33]. Remarkably, the Wzy-dependent Oag biosynthetic pathway contains all of these: Wzx, a flippase that flips the OS-linked undecaprenyl pyrophosphate (Und-PP) carrier lipid across the lipid bilayer creating lipid asymmetry; Wzy, a polymerase that modifies the flipped Und-PP carrier lipid by attaching multiple OSs to form the RUs of the Oag; and a copolymerase that assembles into a large homo-oligomeric structure creating high local protein concentrations on the membrane surface. Thus, it is possible, if not likely, that the Wzy-dependent Oag biosynthetic pathway can deform the bacterial inner membrane. Steric clashes and electrostatic repulsion between adjacent FepE protomers within the complex could generate enough lateral pressure within the plane of the membrane to cause its deformation. It has been suggested that multiple short Und-PP lipids linked to Oag could bind inside the periplasmic domain simultaneously [20]. Thus, it is possible that the recruitment of multiple Und-PP lipids linked to Oag RUs during the polymerization process at the inner membrane could be creating an asymmetry. The high local concentrations of FepE and Und-PP lipids on the outer leaflet of the inner membrane could work together to deform the inner membrane. Although, to our knowledge, membrane deformation has not been discussed previously with regard to the Wzy-dependent biosynthetic pathway, the split-ring structures seen here could be the first clues of its possibility.

### Variability analysis reveals a highly dynamic FepE complex

**Fig. 4.**
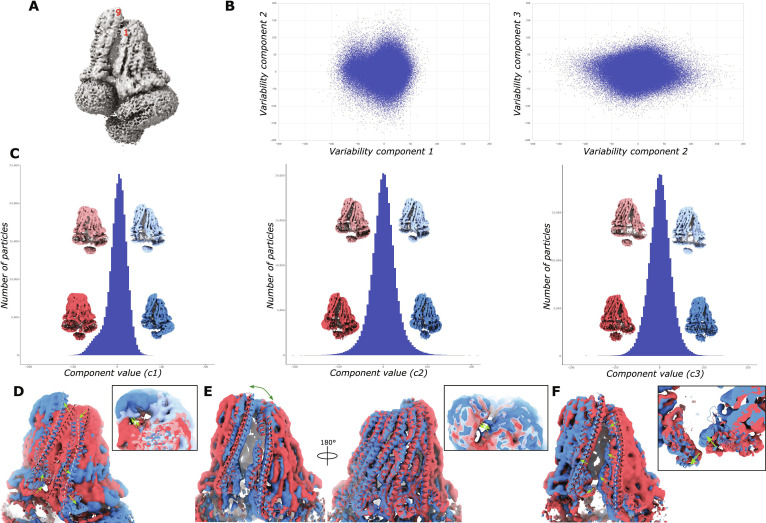
Dynamics of the nonameric FepE complex. (A) Cryo-electron microscopy (Cryo-EM) density map of a consensus 3-dimensional (3D) refinement of all particles containing 9 protomers. (B) Scatter plots of individual particles displaying the extent of variability across the 3-component system solved using cryoSPARC’s 3D variability analysis algorithm (3DVA). (C) Histograms showing the distribution of latent particle coordinates across each component with corresponding negative (red) and positive (blue) density maps at representative points along the component value. (D) Component 1 resolves the opening of the FepE complex with α6 of the ninth protomer ratcheting upward while simultaneously the first protomer twists downward. Inset: overlay of density maps showing dynamics in the region of the L4 loop. (E) Component 2 resolves a large scissoring movement of an open nonameric complex. Acting as the joint, only limited movement is observed of the central protomer 5 at the back of the complex compared to protomers 1 and 9 at the front that can be seen closing. Inset: overlay of density maps showing dynamics in the region of the L4 loop. (F) Component 3 resolves protomer 1 twisting inward toward the center of an open nonameric complex while protomer 9 swings slightly outward. Inset: zoom of the dynamics. For easier visualization, FepE cartoons of protomers are overlaid with the same 3D features of the density maps. The movements described here are visualized in Movie S1.

The missing cryo-EM density of the L4 loop and transmembrane domain of the last and first protomers, respectively, combined with considerably lower local-resolution estimates in parts of the open FepE complexes, particularly the first and last protomer (Fig. S2), hint at a potentially highly dynamic complex. In an attempt to visualize any potential movements of the FepE complex directly in the cryo-EM data, we performed a principal component analysis using cryoSPARC’s 3-dimensional (3D) variability analysis algorithm (3DVA) [34] on FepE complexes containing 9, 8, and 7 protomers (Fig. 4, Figs. S5 and S6, and Movies S1 to S3). Remarkably, this analysis revealed, beyond the simple fluctuation typically observed in rigid structures, large continuous flexibility across the FepE complex as well as discrete movements of domains and helices that could be mechanistically important.

Starting from a consensus refinement of nonameric particles (Fig. 4A), 3 components (Fig. 4B) were solved using 3DVA. The 2-dimensional (2D) scatter plots (Fig. 4B) indicate the extent of the variability along each dimension. The slight bimodal spread along component 1 (Fig. 4B left) corresponds to a ratcheting between the open and closed conformations of the nonameric complex, while the broad spreading along component 2 (Fig. 4B right) corresponds to large scissoring and flexing motions of the open conformation. Although using standard 3D classification (Fig. S1) we were able to distinguish between open and closed nonameric particles, 3DVA enabled the visualization of intermediates between the 2 conformations (Fig. 4C left). Additionally, a symmetric scissoring (Fig. 4C middle) and the discrete swinging of the first and last (Fig. 4C right) protomers of the open nonameric complex can be visualized. The opening of the complex can be best described as a ratcheting backward of protomer 9 together with the ratcheting and twisting downward of protomer 1 (Fig. 4D), resulting in a skewed open complex described above. The bottom of the long α6 appears to be the pivot point for the ratcheting backward of protomer 9 with the top bending backward while the small 3-helix bundle (α3 to α5) swivels slightly upward. The bending backward of the top of α6 is similar to the movement of the alternating α6 and L4 domain of WzzE [17]. Upon opening, a broad scissoring motion occurs (Fig. 4C middle and Fig. 4E) characterized by large displacements of the terminal protomers; meanwhile, protomer 5 acts as the scissor joint, exhibiting little movement (Fig. 4E right). Additionally, a discrete turning of protomer 1 inward toward the center of an open nonameric complex is accommodated by a slight swing outward of protomer 9 (Fig. 4C right and Fig. 4F). Mutations to the single residue L168 [15] (located at the base of the periplasmic base at the start of the L3 loop that interacts with the α2 of an adjacent subunit) resulted in considerably shortened Oag molecules, consistent with this region being important in controlling the correct dynamics of the complex.

Remarkably, in addition to the ratcheting and scissoring movements described above, 3DVA also reveals dynamics of the L4 loop located at the top of the periplasmic domain. Although it is not required for correct oligomerization [19], it has been suggested that the flexibility of the L4 loop could mediate conformational changes associated with function [14,19]. Indeed, since the periplasmic domain is narrowest at the top, L4 loops can interact with multiple protomers around the complex; any potential conformational changes to the L4 loops could have dramatic effects on the full FepE complex. Consistent with that, 3DVA revealed changes in the cryo-EM density corresponding to the L4 loop during the opening/closing and scissoring of the complex (insets of Fig. 4D and E and Movie S1). Although resolution limits in this area prohibit a detailed analysis of the interaction, conformational changes to the cryo-EM density attributed to the L4 loops can be seen around the entire top of the periplasmic domain. In particular, the density attributed to the L4 loop of protomer 9 of the open nonameric state can be seen reaching across the opening to interact with protomer 1 (insets of Fig. 4D and E and Movie S1) and potentially other nearby protomers. As suggested by Kalynych et al. [14,19], the conformational changes of the L4 loops seen here could be mediating larger conformational changes within the full FepE complex, underscoring their essential role in the production of long polysaccharide chains.

3DVA of the 7- and 8-protomer complexes resulted in very similar movements to the 9-protomer complex, but to a much lesser extent (Figs. S5 and S6 and Movies S2 and S3). For example, component 1 of the 7-protomer complex displays a smaller but similar ratcheting-like movement as seen in the opening of the nonameric complex (Fig. S5 and Movie S2). Similarly, the broad spreading along component 1 of the 8-protomer complex (Fig. S6A left and Movie S3) corresponds to large scissoring and flexing as seen in component 2 of the nonameric complex. Although the dynamics observed here are in the absence of the polymerase Wzy, none of the observed movements would necessarily exclude its presence. Even at its smallest, most constricted size, there would be sufficient space within the FepE membrane chamber to contain Wzy. The presence of Wzy interacting with FepE could have a stabilizing effect that could reduce the magnitude of the movements. However, the similar movements seen across all the oligomeric states could suggest that these movements are a robust feature of FepE.

### LPS chain lengths are modulated by the copolymerase periplasmic volume size

**Fig. 5.**
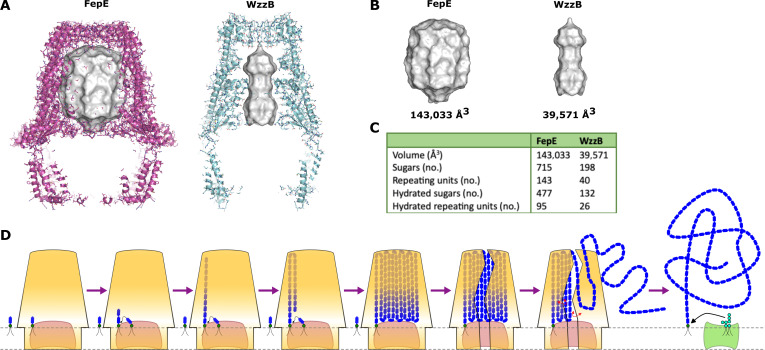
Proposed mechanism of O-antigen chain length modulation. (A) Measurement of copolymerase periplasmic volumes. The closed complexes of FepE and WzzB (Protein Data Bank [PDB] ID 6RBG) sliced to display the interior periplasmic volumes. Gray surfaces represent the volume of the periplasmic domain as calculated using the CavitOmiX plug-in in PyMOL. (B) The periplasmic domain volume of FepE is 3.6 times larger than that of WzzB. (C) Table comparing the periplasmic domain volumes of FepE and WzzB with the maximum number of sugars that they could hold. See Materials and Methods for how the values are calculated. (D) Proposed O-antigen chain length modulation. The polymerase (salmon colored) and copolymerase (yellow) form a complex with oligosaccharides linked to undecaprenyl pyrophosphate (Und-PP) carrier lipids (green) and subsequently to the nascent polysaccharide chain (blue) growing on the inside of copolymerase’s large periplasmic domain. Once the internal capacity of the copolymerase’s periplasmic domain has been reached, the copolymerase complex is forced open, allowing the exit of the completed polysaccharide chain. Copolymerase dynamics likely aid in the exiting of the polysaccharide chain (red arrows). The completed chain is then available for ligation to the lipid A-core (cyan) by WaaL (light green), completing the lipopolysaccharide (LPS) molecule.

Despite overall similar exterior complex dimensions, at the protomer level, many small adjustments of loops and helices add up to make the interior diameter of the FepE complex nearly double compared to that of the WzzB complex, transforming FepE into a large barrel. This results in a periplasmic domain volume of 143,033 Å^3^ for FepE, which is 3.6 times larger than that of WzzB at 39,571 Å^3^ (Fig. 5A and B). Since it is known that FepE is responsible for the chain length modulation of very long LPS molecules of >80 RUs in length compared to ~20 in WzzB, this large difference in periplasmic volumes suggests that there could be a relationship between the 2. Using a conservative volume of 300 Å^3^ for hydrated sugars, the FepE periplasmic domain could contain a maximum of 477 hydrated sugars, or 95 hydrated RUs (Fig. 5C). Similarly, the WzzB periplasmic domain could hold a maximum of 132 hydrated sugars, or 26 hydrated RUs (Fig. 5C). The calculated values match extremely well the experimentally determined LPS chain lengths modulated by FepE [14,15] and WzzB [12], suggesting that polysaccharide chain length is controlled by the volume of the polysaccharide copolymerases’ periplasmic domain and implying that sugar chains must grow on the inside of the periplasmic domain. This is consistent with previous biochemical studies that suggested that the internal cavity of FepE is responsible for Oag polysaccharide length control [15,18,35]. Additionally, several mutagenic studies [14,19–22,26] have suggested that surface regions on both the interior and exterior throughout the entire length of the periplasmic domain determine the length of the growing polysaccharide. These seemingly contradictory results suggest that the entire internal periplasmic volume is acting to modulate the polysaccharide length opposed to any one traditional binding site.

## Discussion

The new data and analysis presented here suggest a remarkably simple mechanism for the modulation of the length distribution of the synthesized polysaccharide chain (Fig. 5D). Simply, the polysaccharide chain grows on the inside of copolymerase’s large periplasmic domain, with its length being determined by the internal volume of the domain. Once the internal capacity of the copolymerase’s periplasmic domain has been reached, the copolymerase complex is forced open to allow the exit of the completed lipid-linked polysaccharide chain. Although the exact oligomeric state and conformation of FepE when in complex with the polymerase Wzy is currently unknown, this mechanism suggests that it must be the nonameric form in order to correctly modulate Oag chain length. This mechanism is comparable to a mechanism proposed by Huszczynski et al. [35] that suggests that when the maximum internal capacity of the copolymerase has been filled, the complex disassembles. Instead of the complete disassembly, our data suggest that the complex opens to allow the release of the completed polysaccharide chain. Additionally, the split-ring conformations of the open complexes suggest that membrane distortion could be associated with FepE. This could suggest that the mechanism of Oag chain length modulation shares aspects seen in mechanosensitive ion channels that open and close in response to mechanical stress via increased tension in the lipid bilayer [36]. As membrane tension increases in these channels, the closed homo-oligomeric complex tilts to create a large pore [37]. Herein, pressure on the membrane from a densely packed periplasmic domain containing the full-length Oag polysaccharide could have an analogous effect, causing the copolymerase complex to tilt and open up. The mechanistic similarities between these 2 seemingly unrelated classes of proteins hint at an underlying fundamental biological mechanism.

Finally, this simple mechanism can potentially explain many of previous biochemical, mutational, and cross-linking studies [14,15,19–22,26,35]. The seemingly contradictory mutations to both interior and exterior surface residues that alter the Oag chain length can then be rationalized, as they likely weaken the integrity of the complex, causing it to split open before a full-length polysaccharide chain can be achieved. As suggested by Huszczynski et al. [35], this is consistent with the additional protomer–protomer interactions described above providing increased stability to the FepE complex, allowing for the elongation of very long Oag chains. These mutations are also likely altering the dynamics of the complex; for example, the mutation of selected residues to alanines and valines had no effect on LPS length [18], compared to the mutation of the same residues to cystines to create subunit–subunit cross-links that resulted in shortened LPS molecules [15]. We resolved the opening of the nonameric FepE complex (Movie S1), confirming that conformational changes to the essential L4 loops occur during this process [14,19]. This would again be consistent with these loops mediating the opening/closing of the complex and consistent with the need for a negatively charged residue at amino acid position 268 [15]. Additionally, other copolymerase dynamics revealed by 3DVA, in particular the scissoring and discrete swinging of protomers of the open nonameric complex, likely aid in the ushering out of the completed polysaccharide chain, explaining previous studies that indicate that conformational changes are required for the release of the Oag-linked Und-PP chains [14,19]. The structures and dynamics presented here suggest that polysaccharide copolymerases act as a highly tuned molecular machine, striking a perfect balance of stability that allows the periplasmic domain to fill completely while still remaining dynamic enough to open and release completed Oag polysaccharides.

## Materials and Methods

### Protein expression and purification

The full-length FepE gene was amplified from *E. coli* K12 genomic DNA using standard polymerase chain reaction techniques using the primers EcFepE_fwd and EcFepE_rev (Table S3) and inserted into a modified pWaldo vector that replaces the green fluorescent protein reporter with a C-terminal 8×His tag using the restriction sites *XhoI* and *EcoRI* and their sequences confirmed by DNA sequencing. Large-scale protein expression, solubilization with *n*-dodecyl-β-d-maltoside, and purification was performed as described for WzzB [16] and WzzE [17].

### Cryo-EM sample preparation and data collection

Purified protein at 3.0 mg/ml (3 μl) was applied to cryo-EM grids (Quantifoil 2/2 Au 300) previously glow-discharged under vacuum for 40 s at 20 mA (PELCO easiGlow), incubated for 30 s, blotted for 3 s, and plunge frozen in liquid ethane using a Vitrobot Mark4 grid freezing device (FEI) with the chamber maintained at 4 °C and 100% relative humidity as previously described [16]. An optimized grid was imaged with a Thermo Scientific Titan Krios G3 electron microscope equipped with a K3 camera, operating at 300 kV. Movies of 40 frames each were acquired in electron counting mode with a total exposure dose of 48 electrons/Å^2^ and stacked into a single Medical Research Council (MRC) stack using the EPU automatic data collection control software. Detailed data collection parameters can be found in Table S1.

### Cryo-EM image processing

Motion correction was performed with PatchMotion, and contrast transfer function parameters were estimated from averaged movies using PatchCTF in cryoSPARC v4 [38]. Automatic particle selection was performed with templates from the initial 2D classification using manually picked particles. The number of particle images were reduced by 2D and 3D classifications (Fig. S3). Initial maps were calculated ab initio, and the final maps were refined using cryoSPARC’s nonuniform refinement feature [39] with and without the application of C9 symmetry where applicable. Detailed image processing parameters can be found in Table S1; 3D variable analysis [34] was performed using particles from consensus refinements, using a filter resolution of 4.5 Å and solving for 3 components.

### Model building and refinement

A single copy of a FepE model generated by AlphaFold3 [40] was docked and refined against the 2.8-Å-resolution, C9-symmetrized map. Once docked, the model was further improved through iterative rounds of refinement with phenix_real_space_refine [41] and fixing with Coot [42]. After the asymmetric unit was completed in this way, C9 symmetry was applied to the model with the Phenix suite of programs [41]. This full complex was then refined against the C9-symmetrized map with phenix_real_space_refine and further improved through iterative rounds of refinement and fixing with Coot. The open conformations of the FepE complex were built in a similar way: single protomers from the closed nonameric FepE complex were docked to the respective C1 maps followed by improvements through iterative rounds of refinement and fixing with Coot. The final models were validated with MolProbity [43]. All figures containing macromolecular structures were made with UCSF ChimeraX [44] or PyMOL [45]. Detailed model building statistics can be found in Table S1.

### Volume calculations

The interior volume of the periplasmic domain was calculated using the CavitOmiX plug-in (Innophore GmbH) in PyMOL using a grid spacing of 1.0. The cavities were calculated using a modified LIGSITE algorithm [46]. Residues 56 to 289 and 65 to 330 for WzzB (Protein Data Bank ID 6RBG) and FepE were used, respectively, to define the periplasmic domain. The interior volumes of the FepE and WzzB structures were calculated with different sphere radii ranging from 1.4 Å, corresponding to an isolated water molecule [47], with the sphere radius stepwise increasing up to 5.4 Å, which for FepE resulted in volumes of 1.67 × 10^5^ and 1.43 × 10^5^ Å^3^, respectively, whereas for WzzB the volumes were smaller, being 6.96 × 10^4^ and 3.96 × 10^4^ Å^3^, respectively, for the smallest and largest sphere radii. The Oag polysaccharide in *E. coli* K12 is not expressed due to a mutation in the rfb gene cluster, but the expression of the polysaccharide can be restored by a plasmid carrying part of the rfb mutation of K12 [48]. A constructed strain with all *rfb* genes intact synthesized a variant of the Oag of serogroup O16 having a pentasaccharide RU with 4 sugars in the backbone of the RU and 1 sugar as a side-chain residue [49]. To estimate the number of RUs of a polysaccharide that may be accommodated within the interior of the periplasmic portion of the Wzz, the volume of a hydrated monosaccharide can be estimated from its density and molecular mass (1.562 g·cm^−3^ and 180.16 g·mol^−1^ for d-glucose, respectively) [50] in conjunction with the Avogadro constant (6.022 × 10^23^ mol^−1^), resulting in a volume of ~200 Å^3^ for a d-glucose molecule and the fact that one water molecule (in bulk water being 29.7 Å^3^) [51] per hydroxyl group is associated with the sugar as deduced by hydrodynamic modeling [52]. As part of a polysaccharide, this would amount to about 3 water molecules per sugar residue in the polymer; i.e., out of the 5 hydroxyl groups in a hexose like d-glucose, 2 of them would be linked as a glycoside in a polysaccharide, leaving 3 hydroxyl groups of each sugar residue to be hydrated in the polymer, resulting in a volume of ~300 Å^3^ for a hydrated monosaccharide entity. With 5 sugar residues per RU, the number of RUs that may be located within the FepE structure is ~110 or ~95 for the smallest and largest sphere radii, respectively, whereas for the WzzB structure, it is ~45 or ~25 RUs. This calculation is corroborated by LPS modal distributions of Oags in LPS experimentally determined by matrix-assisted laser desorption/ionization–mass spectrometry being ~23 and ~24 RUs for *E. coli* serogroups O159 [53] and O164 [54], respectively.

## Data Availability

The cryo-EM density maps and atomic coordinates have been deposited in the Electron Microscopy Data Bank and Protein Data Bank under the accession codes EMD-54965, EMD-54966, EMD-54967, EMD-54968, and EMD-54969 and 9SKI, 9SKJ, 9SKK, and 9SKL, respectively. All relevant data supporting the key findings of this study are available within the article and its Supplementary Materials files or from the corresponding authors upon reasonable request. Additional maps and source data are available from the corresponding authors upon reasonable request.
