# Supplementary material for: Structural Basis of Lipopolysaccharide O-Antigen Chain Length Modality
Source: Research (Wash D C). 2026 May 12;9:1276. doi: 10.34133/research.1276 (PMC13161533; doi:10.34133/research.1276)
Supplement: Supplementary 1 — Figs. S1 to S6 Tables S1 to S3 Movies S1 to S3 [file research.1276.f1.zip › Wiseman_elal_FepE_science_supplementary_materials_Research_revisions.pdf]

Supplementary Materials for

Structural basis of lipopolysaccharide O-antigen chain length modality

Benjamin Wiseman[1*], Göran Widmalm[2] and Martin Högbom[1*]

[1]Department of Biochemistry and Biophysics and Science for Life Laboratory, Stockholm University; Stockholm, Sweden.

[2]Department of Chemistry, Stockholm University; Stockholm, Sweden.

* Correspondence: BW, benjamin.wiseman@dbb.su.se, MH, hogbom@dbb.su.se

This PDF file includes:

Figs. S1 to S6
Tables S1 to S3

Other Supplementary Materials for this manuscript include the following:

Movies S1 to S3

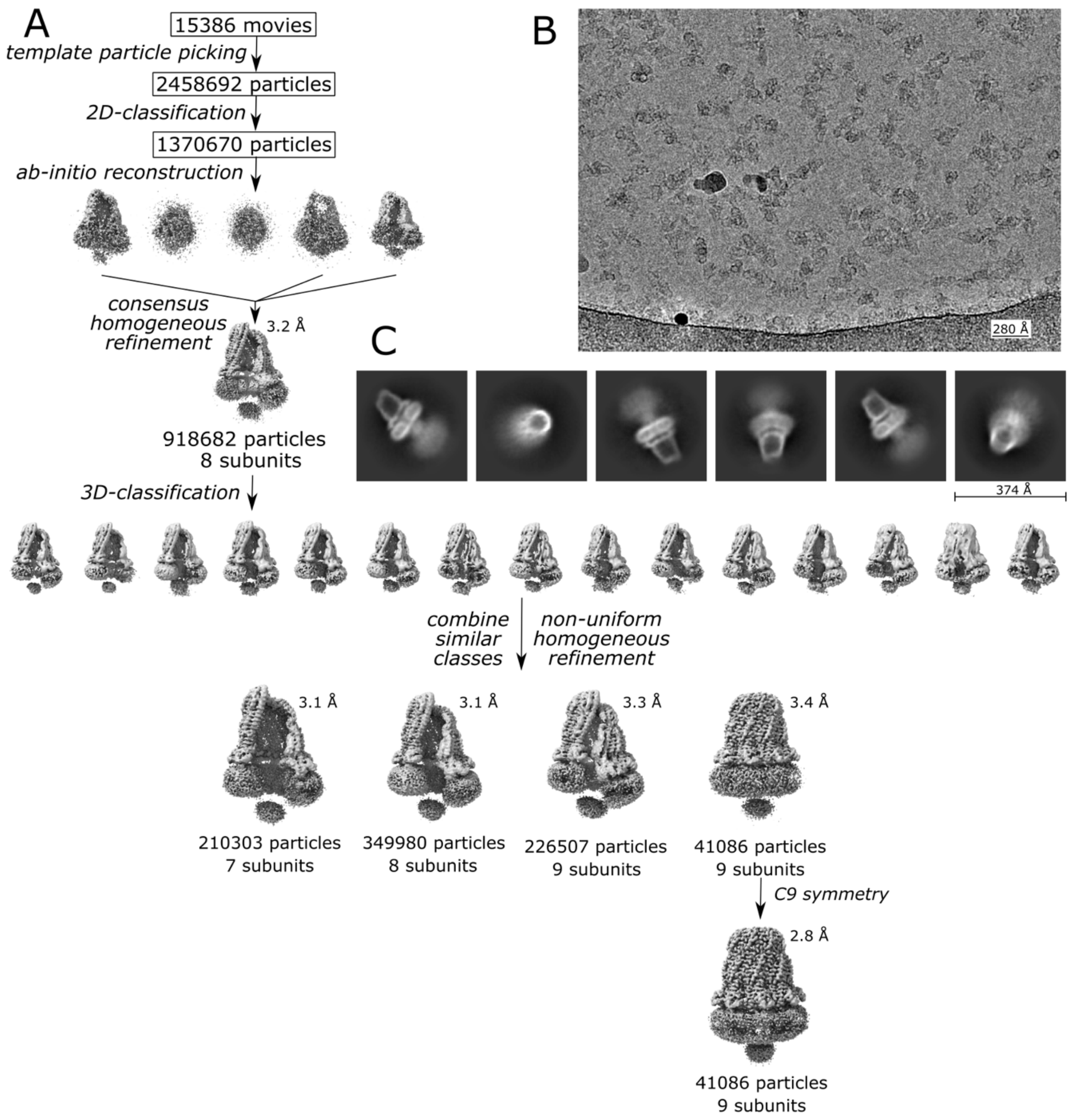

Fig. S1. Cryo-EM particle processing workflow of FepE. (A) Particle processing. After a single round of 2D-classification, particles were further classified by reference-free *ab-initio* 3D classification. Particles from similar volumes were pooled and refined into an initial consensus volume containing 8 FepE subunits. Starting from this initial consensus refinement, 3D-classification identified unique classes containing 7, 8 and 9 FepE subunits. Each class was refined using cryoSPARC's non-uniform homogeneous refinement and further improved with per-particle CTF refinement, and the application of symmetry where applicable. (**B)** Typical micrograph from a single data collection of 15386 micrographs used for automatic particle picking for the 2D classification. (**C)** 2D class averages of FepE.

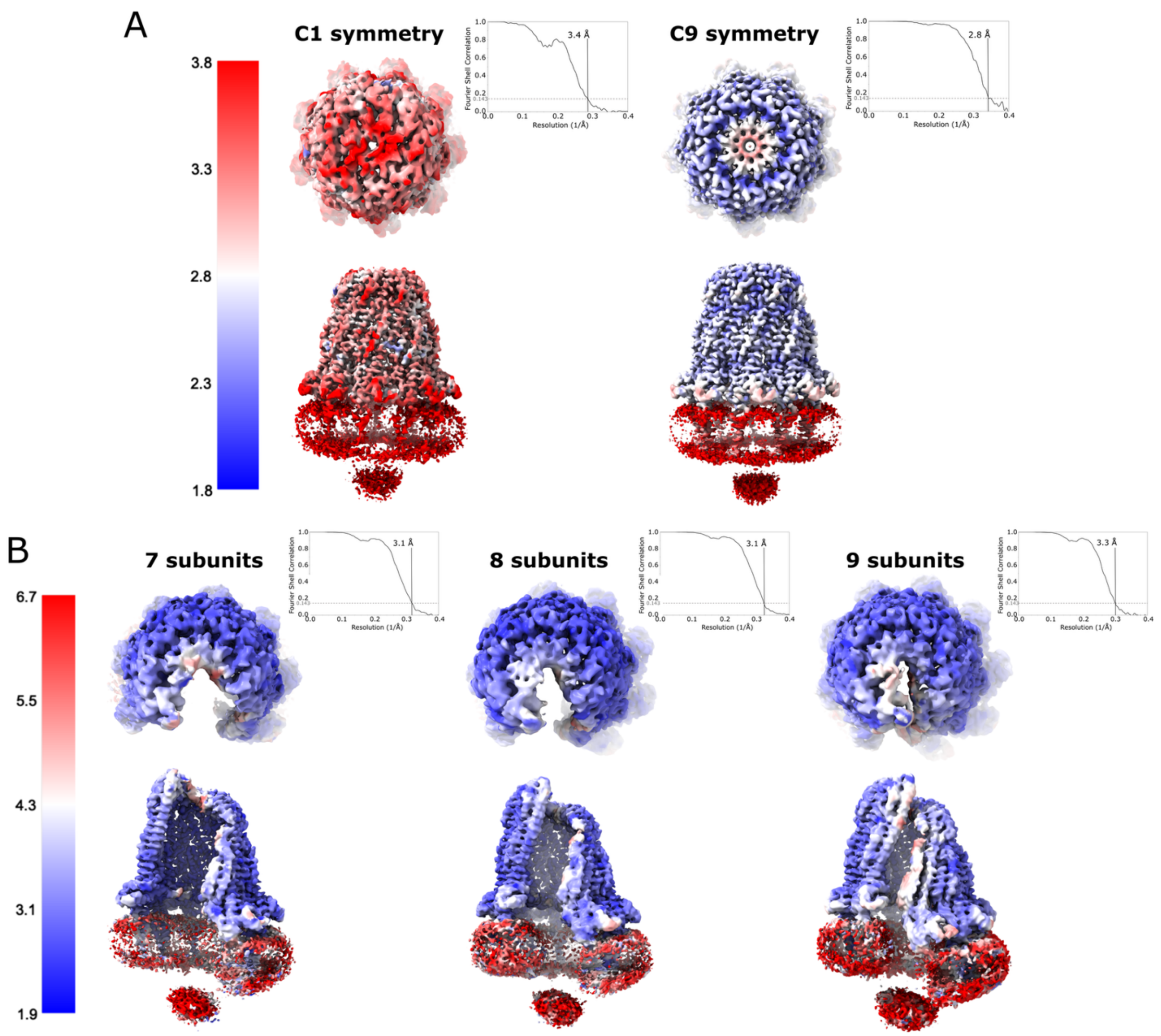

Fig. S2. Cryo-EM data assessment of FepE. (**A**) Local-resolution estimation and Fourier Shell Correlation (FSC) (inset) of the C1, and C9 symmetrized density maps of the closed nonameric form. **(B)** Local-resolution estimation and Fourier Shell Correlation (FSC) (inset) of the open forms containing 7, 8, and 9 FepE subunits.

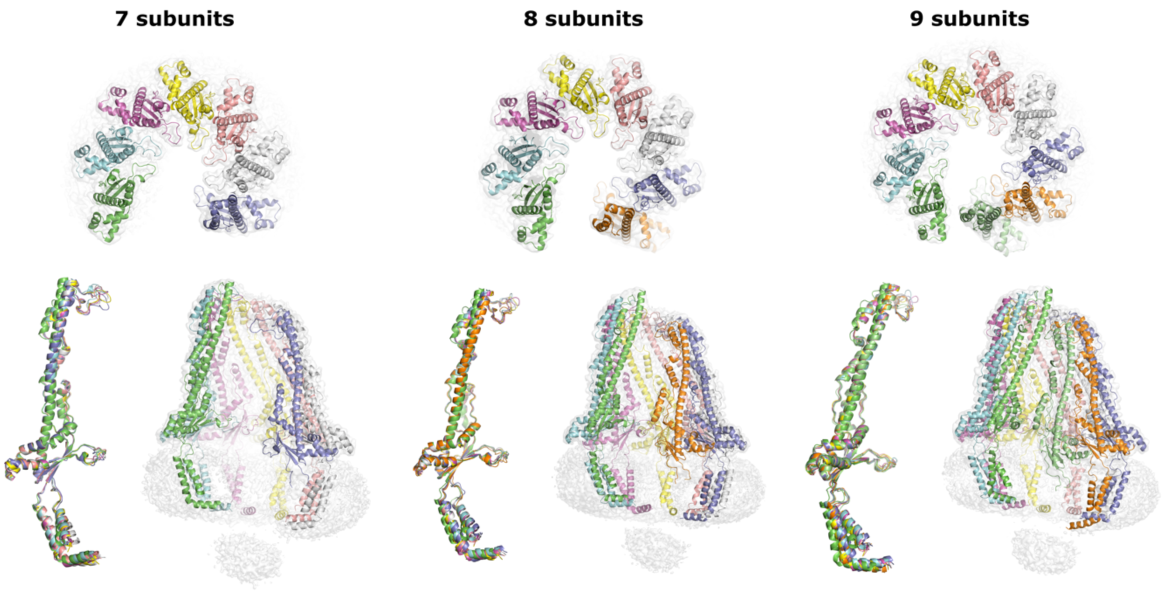

Fig. S3. Overall structure of the open forms of FepE. Cartoon representation of the open forms of the FepE complex overlaid with their corresponding density map (white). Top: view from the top sliced to the level of the interior L3 loop. Bottom left: structural alignment of the protomers within each oligomeric state. See table S2 for R.M.S.D. values. Type or paste caption here. Create a page break and paste in the figure above the caption.

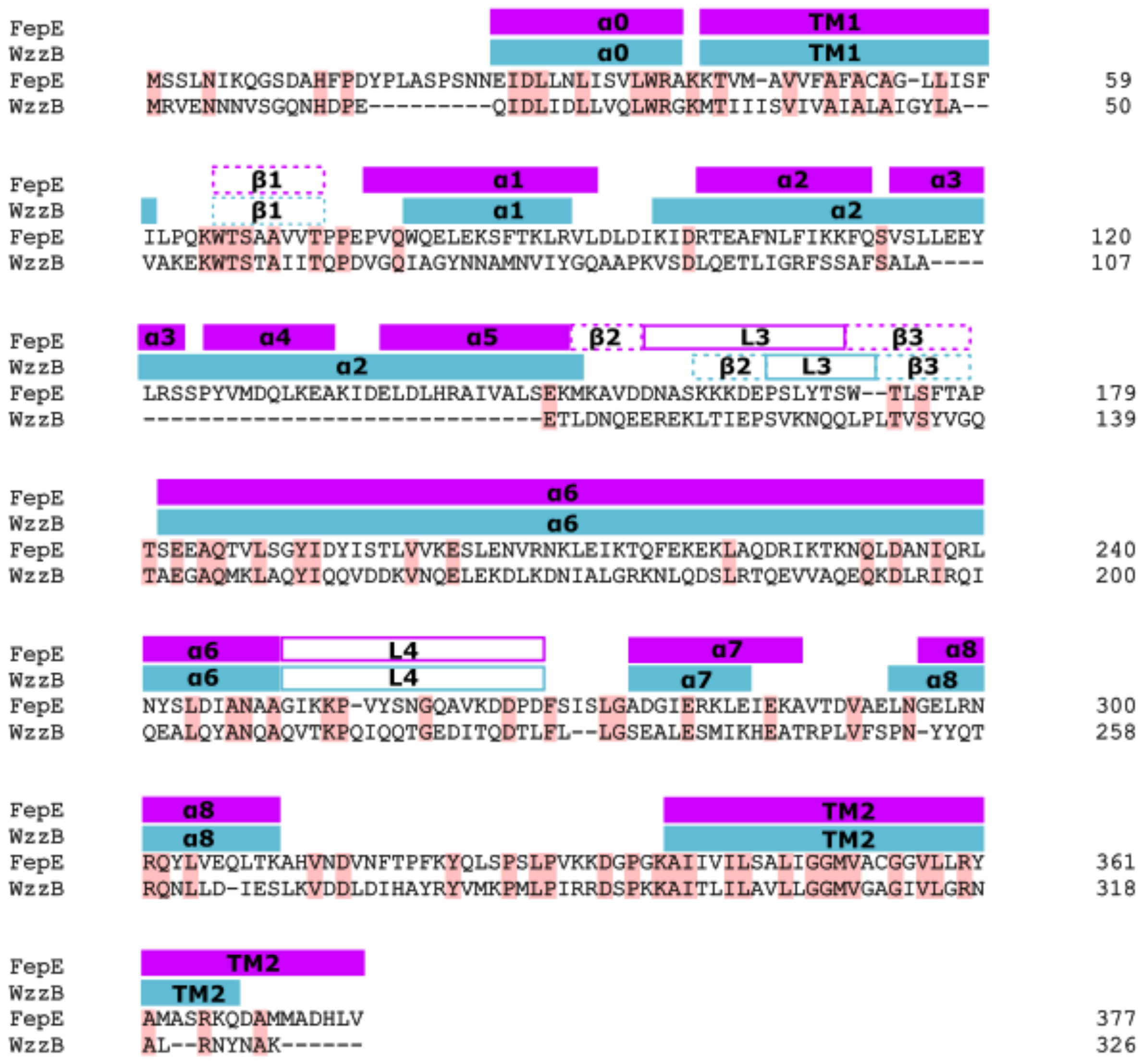

Fig. S4. *E. coli* FepE : WzzB amino acid sequence alignment. Magenta and cyan bars and boxes represent the location of β-sheets, α-helices and interior loops for FepE and WzzB respectively. The red highlighted residues represent conserved residues.

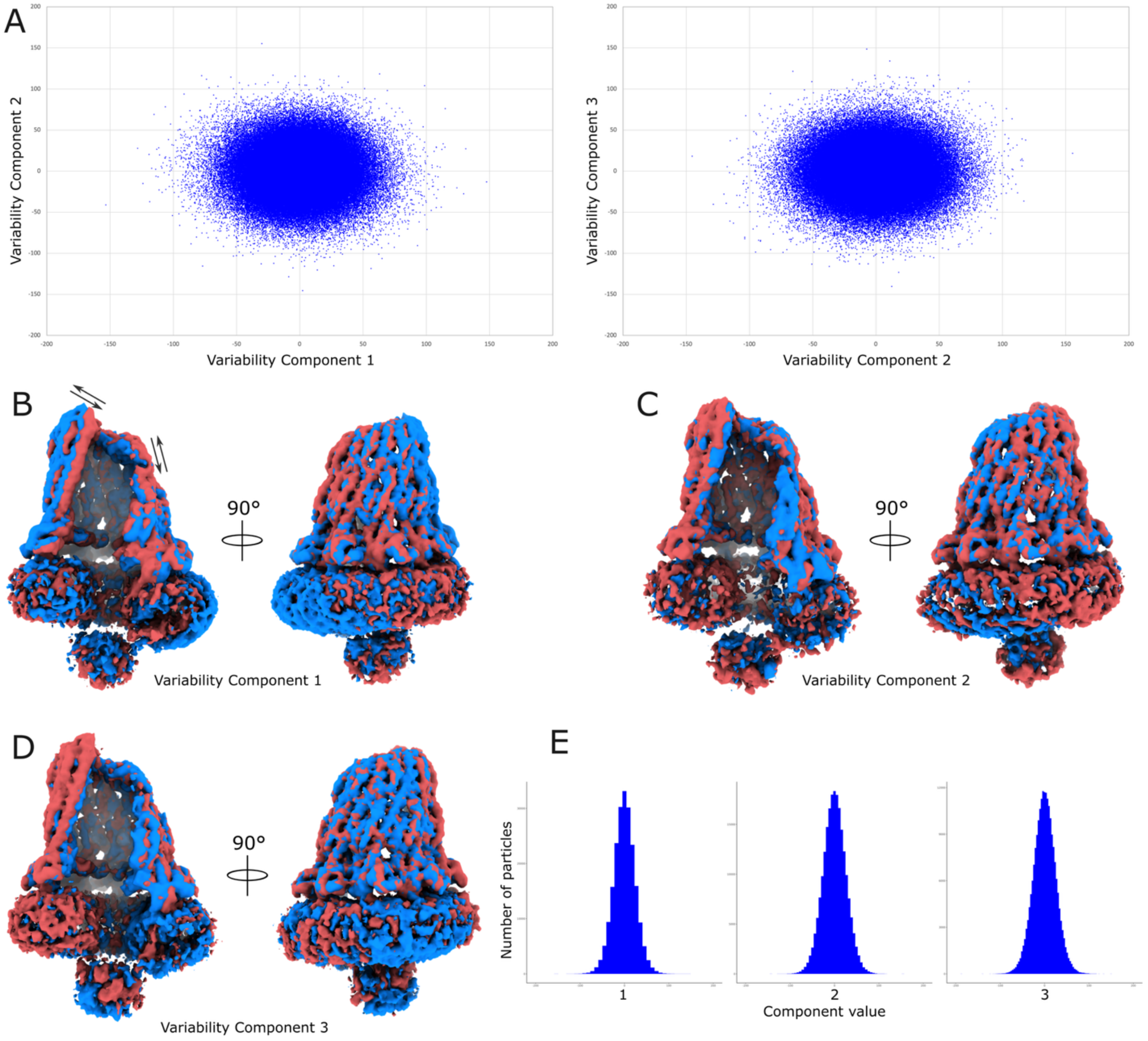

Fig. S5. 3D variable analysis of the 7-protomer oligomeric state of FepE. (A) Scatter plots of individual particles displaying the extent of variability across the 3-component system. **(B), (C) and (D)** Negative and positive density maps of the three components. Component 1: a slight bending upwards of α6 of the 7th protomer while simultaneously the 1st protomer twists downwards. The movement is analogous to what is seen in component 1 of the nonameric complex, but to a much smaller extent. Components 2 and 3: a simple flexing typically observed in rigid structures, in addition to a slight swinging or flexing of the end protomers 1 and 7 is observed. (**E**) Histograms showing the distribution of latent particle coordinates across each component. The movements described here are summarized in Movie S2.

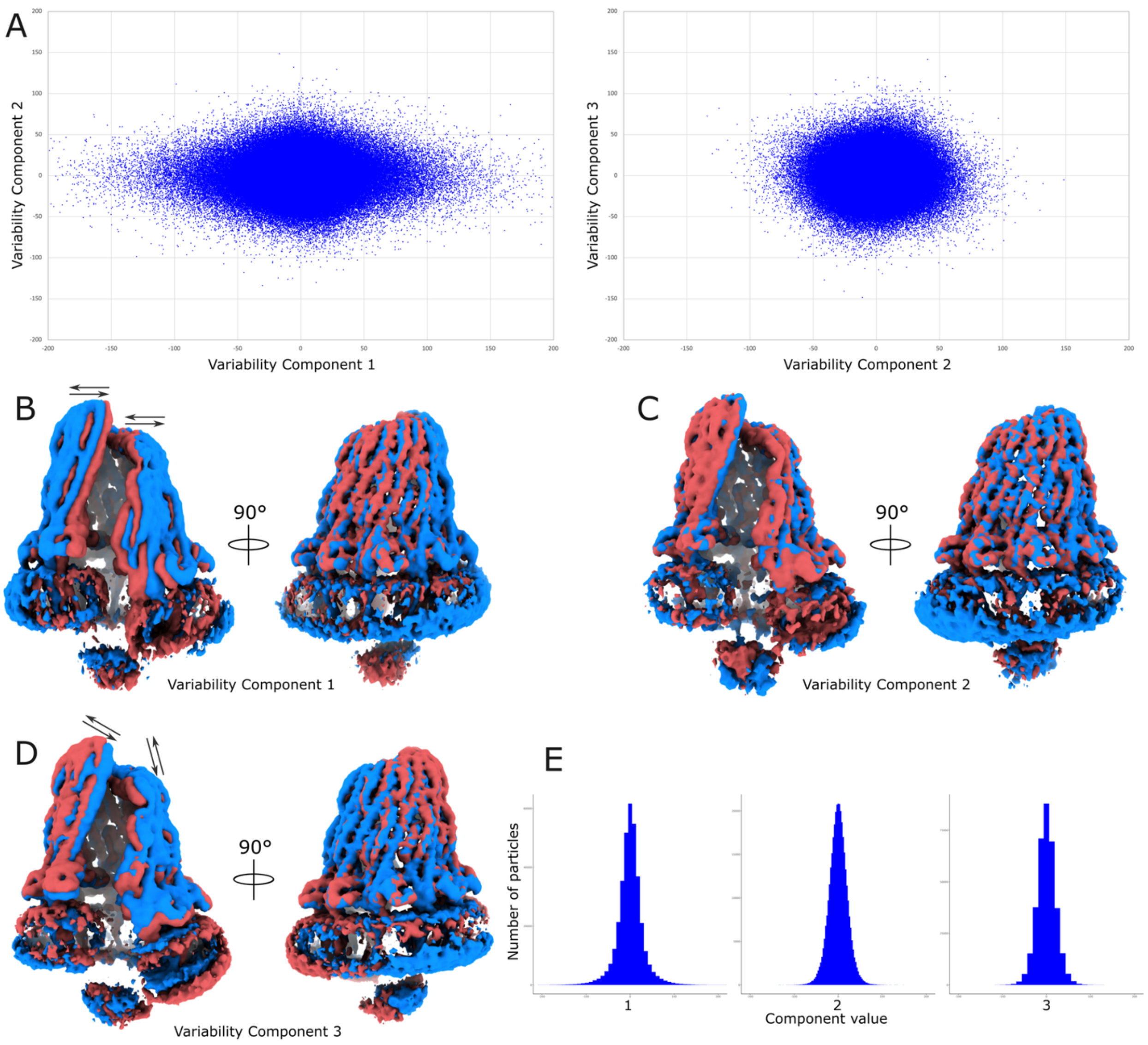

Fig. S6. 3D variable analysis of the 8-protomer oligomeric state of FepE. (A) Scatter plots of individual particles displaying the extent of variability across the 3-component system. **(B), (C)** and **(D)** Negative and positive density maps of the three components. Component 1: a slight bending upwards of α6 of the 8th protomer while simultaneously the 1st protomer twists downwards. The movement is analogous to what is seen in component 1 of the nonameric complex, but to a much smaller extent. Components 2 and 3: a simple flexing typically observed in rigid structures, in addition to a slight swinging or flexing of the end protomers 1 and 7 is observed. **(E)** Histograms showing the distribution of latent particle coordinates across each component. The movements described here are summarized in Movie S3.

Table S1. Cryo-EM data collection, refinement and validation statistics.

	**Open oligomeric states**			**Closed oligomeric state**	
	7 subunits<br>EMD-54965<br>PDB: 9SKI	8 subunits<br>EMD-54966<br>PDB: 9SKJ	9 subunits<br>EMD-54967<br>PDB: 9SKK	9 subunits<br>EMD-54968<br>PDB: 9SKL	9 subunits<br>EMD-54969
**Data collection and processing**					
Magnification	105000×	105000×	105000×	105000×	105000×
Voltage (kV)	300	300	300	300	300
Electron exposure (e^{-}/Å^2)	48	48	48	48	48
Defocus range (µm)	0.6 - 1.8	0.6 - 1.8	0.6 - 1.8	0.6 - 1.8	0.6 - 1.8
Pixel size (Å)	0.828	0.828	0.828	0.828	0.828
Extraction box size (pixels)	450	450	450	450	450
Symmetry imposed	C1	C1	C1	C9	C1
Initial particle images (no.)	918682	918682	918682	918682	918682
Final particle images (no.)	210303	349980	226507	41086	41086
Map resolution (Å)	3.1	3.1	3.3	2.8	3.4
FSC threshold	0.143	0.143	0.143	0.143	0.143
Map resolution range (Å)	1.9 - 20	1.9 - 8.9	1.9 - 14	1.8 - 8.9	2.2 - 44
**Model refinement**					
Initial model used (PDB code)	-	-	-	-	
Model resolution (Å)	3.6	3.5	3.7	3.2	
FSC threshold	0.5	0.5	0.5	0.5	
Model resolution range (Å)	1.9 - 5.8	1.9 - 5.4	1.9 - 5.6	1.8 - 3.9	
Map sharpening *B* factor (Å^2)	83.6	89.0	89.6	80.8	
Model composition					
Non-hydrogen atoms (no.)	17689	20288	22904	23391	
Protein residues (no.)	2241	2571	2903	2970	
Ligands (no.)	0	0	0	0	
B factors (Å^2)					
Protein	142	175	197	84.9	
Ligand	-	-	-	-	
R.m.s. deviations					
Bond lengths (Å)	0.004	0.004	0.004	0.004	
Bond angles (°)	0.885	0.905	0.924	0.826	
Validation					
MolProbity score	1.57	1.60	1.65	1.27	
Clashscore	5.54	5.14	5.83	4.23	
Poor rotamers (%)	0.00	0.93	0.75	0.92	
Ramachandran plot					
Favored (%)	96.0	95.4	95.3	97.7	
Allowed (%)	4.0	4.6	4.7	2.30	
Disallowed (%)	0.00	0.00	0.00	0.00	

Table S2. R.M.S.D. between subunits within each oligomeric FepE state.

	R.M.S.D. (Å)*			
**Subunit**	**7**	**8**	**9**	**9****
**1**	0	0	0	0
**2**	0.680	0.865	0.923	0.031
**3**	0.694	0.913	1.030	0.040
**4**	0.636	0.844	0.948	0.036
**5**	0.611	0.821	0.906	0.031
**6**	0.739	0.770	0.856	0.037
**7**	0.900	0.860	0.858	0.039
**8**	-	1.119	1.042	0.034
**9**	-	-	1.418	0.033

*subunits were aligned to subunit 1 of each oligomeric state.
**closed nonameric, C9 symmetric complex.

Table S3. Cloning and mutagenic primers for *E. coli* K12 *fepE*.

Primer Name	Sequence (5' → 3')	Restriction site
EcFepE_fwd	ACTCAGCTCGAGATGTCATCACTGAATATTAAACAGGGAAG	*XhoI*
EcFepE_rev	ATCGACGAATTCAACTAAGTGGTCTGCCATCATG	*EcoRI*

Movie S1.

3D variable analysis of the 9-protomer oligomeric state of FepE. 3D variable analysis was performed using particles from a consensus refinement of FepE particles containing 9 protomers using a filter resolution of 4.5 Å and solving for 3 components.

Movie S2.

3D variable analysis of the 8-protomer oligomeric state of FepE. 3D variable analysis was performed using particles from a consensus refinement of FepE particles containing 9 protomers using a filter resolution of 4.5 Å and solving for 3 components.

Movie S3.

3D variable analysis of the 7-protomer oligomeric state of FepE. 3D variable analysis was performed using particles from a consensus refinement of FepE particles containing 8 protomers using a filter resolution of 4.5 Å and solving for 3 components.
